# Amino acids and energy digestibility in extruded or roasted full fat soybean fed to broiler chickens without or with multienzyme supplement containing protease, phytase, and fiber degrading enzymes

**DOI:** 10.1016/j.psj.2021.101511

**Published:** 2021-09-28

**Authors:** Aizwarya Thanabalan, Mohsen Mohammadigheisar, Elijah G. Kiarie

**Affiliations:** Department of Animal Biosciences, University of Guelph, Guelph, ON N1G 2W1, Canada

**Keywords:** heat processed full fat soybean beans, broiler chickens, feed enzymes, AMEn

## Abstract

Effects of processing and multienzyme supplement (**MES**) on standardized ileal digestibility (**SID**) of amino acids, apparent retention (**AR**) of components and metabolizable energy (**AME**) content in full fat soybean seeds (**FFSB**) were investigated in broiler chickens. The FFSB were either extruded (**EFFSB**) or roasted (**RFFSB**). A nitrogen free diet (**NFD**) was formulated for SID of AA calculation. The FFSB diets contained 20% crude protein with the ratio of corn starch: sucrose: soy oil (sole sources of energy in NFD) kept constant for calculation of AME. The FFSB diets were fed without or with MES containing phytase, protease, and fiber degrading enzymes. All diets had TiO_2_ indigestible marker. A total of 400-dayu-old Ross 708 male chicks were fed a commercial diet to d 13. On d 14, birds were weighed individually and allocated to cages (10 birds/cage, n = 8). Birds had free access to feed and water. Excreta samples were collected on d 18 to 20, and all birds were necropsied on d 21 for terminal ileal digesta samples. There was no (*P* > 0.05) interaction between processing and MES on SID of AA. Birds fed EFFSB had higher (*P* ≤ 0.048) SID of Arg, Ile, Lys, and Met than birds fed RFFSB. Birds fed MES had higher (85.5 vs. 80.8%; *P* = 0.050) SID of Lys than birds fed non-MES diet. There was interaction (*P* ≤ 0.036) between processing and MES on AR of Ca and P; MES improved retention but largely in EFFSB. There was an interaction (*P* = 0.016) between processing and MES on energy utilization such that MES improved AR of GE, AME, and AMEn in RFFSB only. In general, birds fed EFFSB exhibited higher (*P* < 0.01) energy utilization than birds fed RFFSB. In conclusion, lower Lys and energy utilization in RFFSB relative to EFFSB reflected the impact of the processing regimen. Supplemental enzyme improvement on Lys and minerals digestibility in FFSB and energy utilization in RFFSB suggested value in heat processed feedstuffs.

## INTRODUCTION

Soybean meal (**SBM**), a co-product from oil extraction is the predominant soy product available for the feed industry (NRC, 1994, 2012). However, full-fat soybeans (**FFSB**) are also used in animal nutrition. For utility in non-ruminant nutrition, FFSB must be thermally processed to destroy antinutritional factors and increase oil availability while preserving the protein quality. An optimally processed FFSB is a valuable feed ingredient because of high-quality protein and energy. Moreover, FFSB is desirable for high energy density diets as it minimizes or eliminates the post-pellet application of lipids (Waldroup, 1982). There are many processing methods for FFSB such as extrusion, toasting/roasting, micronizing, and jet sploding, among others and the merits and demerits of these methods in terms of economics and impact on the nutritive value have been documented (Simovic et al., 1972; Waldroup, 1982; Mirghelenj et al., 2013; Ravindran et al., 2014a).

Sufficient inactivation of trypsin inhibitors (**TI**) and other heat-labile anti-nutritional factors is critical for optimal utilization of FFSB. However, processing conditions such as the temperature and the pressure applied for the process duration all influence the nutritive value of FFSB (Simovic et al., 1972; Waldroup, 1982; NRC, 1994; Clarke and Wiseman, 2007; NRC, 2012; Ravindran et al., 2014a). Unlike roasting, the extrusion process exposes the material to high temperatures with high pressure and shear force for a relatively short period (Kim et al., 2006). Extruded and roasted FFSB resulted in similar but superior digestibility of crude protein (**CP**), crude fat, and ash relative to raw FFSB in broiler chickens (Lehmali and Jafari, 2019). However, extruded FFSB (**EFFSB**) had a lower protein dispersibility index than roasted FFSB (**RFFSB**), indicating overheating (Batal et al., 2000; Lehmali and Jafari, 2019). In a comparative evaluation, EFFSB had higher digestibility of dry matter (**DM**), gross energy, and nitrogen (**N**) than RFFSB in pigs (Kim et al., 2000). The differences in nutritive value of EFFSB and RFFSB may reflect the impact of thermal processing regimens. However, analyses of 55 SBM samples (CP 44–48%) sourced from major exporting countries showed that utilization of amino acids and energy in poultry was not associated with processing quality checks (TI, urease index and potassium hydroxide [**KOH**] protein solubility) or concentration of CP, but was highly negatively correlated with the concentration of fiber and ash (Ravindran et al., 2014b). These findings suggested that the concentration of fiber and minerals and attendant complexes had a bigger impact on the nutritive value of soy products (NRC, 2012; Ravindran et al., 2014b). Implications of these findings could be much more important for the FFSB as the hull is intact, and reduced energy use from full-fat oilseeds has been suggested to be due to a lower oil availability linked to the oil-encapsulating effect of the cell wall polysaccharides (Meng et al., 2006; Slominski et al., 2006). Moreover, processing protein feedstuffs increases concentration fiber insoluble nitrogen with negative consequences on CP utilization (Mustafa et al., 2001; González et al., 2002; Machacek and Kononoff, 2009).

Exogenous fiber degrading enzymes and protease have been used to improve the nutritive value of FFSB in both broilers (Erdaw et al., 2017a,b) and pigs (Ayoade et al., 2012; Woyengo et al., 2016). However, high phytate levels in soybean have been associated with negative effects on nutrient utilization in monogastric animals (Cowieson et al., 2017; Zouaoui et al., 2018). A multienzyme preparation (MES) containing phytase, protease and fiber degrading and debranching enzymes has been shown to be effective in improving utilization of nutrients in poultry fed SBM based diets (Ward et al., 2014; Jasek et al., 2015; Ward et al., 2015, 2020) and pigs (Kiarie et al., 2020). The objective of the present study was to investigate the effects of processing method and multienzyme supplement (**MES**) containing protease, phytase, and fiber degrading enzymes on standardized ileal digestibility (**SID**) of amino acids, apparent retention (**AR**) of components, and metabolizable energy (**AME**) content in full fat soybean seeds (**FFSB**) fed to broiler chickens.

## MATERIALS AND METHODS

Animal care and use protocols were approved by the University of Guelph Animal Care and Use Committee. Birds were cared for in accordance with the Canadian Council on Animal Care guidelines (CCAC, 2009).

### Soybean Products and Diets

The extruded sample was procured from Huber farms heritage meats (Kenilworth, ON, Canada). A single-screw autogenous extruder with a processing capacity of 900 kg/h powered by a 150 HP tractor (Insta-Pro 2000, Insta-Pro International, Grimes, IA) was used. The extruder was equipped with a steam-lock ring and die plate with 3 round openings of 1.11 cm in diameter. The beans were initially subjected to 121 to 127°C then 135 to 140°C at the exit. The product transit time averaged 30 ± 1 S, with an average barrel pressure of 31 ± 2 kg/cm^2^. Samples for roasted FFSB were procured from a local farm (James Valley Colony, MB, Canada) that uses an automatic electric-powered 32-ft long, 14-inch diameter stainless steel roaster (Dilts-Wetzel Manufacturing Co., Ithaca, MI). Briefly, the beans are cooked at between 118 and 120°C for 1 h, then to a steeping chamber and continue to be cooked by steam for an additional half hour, exiting the steeping chamber at 121°C. Air-dried EFFSB and RFFSB were ground through the hammer mill before feed manufacture.

A nitrogen free diet (**NFD**, Table 1) was formulated to estimate endogenous nitrogen losses to calculate the SID of amino acids (Adeola et al., 2016). The FFSB diets (EFFSB and RFFSB; Table 1) were designed to contain 20% CP, and the ratio of corn starch to sucrose to soy oil (the sole sources of energy in NFD) was maintained constant to allow the determination of AMEn in FFSB samples using the substitution method as described by Mwaniki and Kiarie (2018). The FFSB diets were fed without or with a MES containing protease, phytase and fiber degrading enzymes (Victus, DSM Nutritional Products Inc., Parsippany, NJ). The target activities for phytase, protease, xylanase, β-glucanase were 2,200 FYT/kg, 8,300 PRO/kg, 600 U/kg, and 200 U/kg of feed, respectively. In addition, mass spectrophotometry proteomic evaluation of the MES confirmed the presence of a wide range of side activities such as arabinofuranosidase, exoarabinase, pectin lyase, pectin methylesterase, and others considered important in facilitating the accessibility of complex substrates to the main enzyme activities (Ward et al., 2020; Ward, 2021). Minerals and vitamins were added to all diets to meet or exceed nutrient requirements (Aviagen, 2014). All the diets contained TiO_2_ (0.50%) as an indigestible marker and were fed as mash.

**Table 1:** Composition of experimental diets, as fed basis.

	N-free	FFSB^1^
Corn starch	77.2	35.1
Full fat soybean-extruded	-	-
Full fat soybean-roasted	-	55.0
Cellulose	5.00	-
Sucrose	8.25	3.75
Soy oil	2.00	0.91
Limestone	1.27	1.24
Mono-calcium phosphate	2.30	1.56
Sodium chloride	0.38	0.35
Sodium bicarbonate	0.04	0.04
Potassium carbonate	1.77	0.30
Titanium dioxide	0.50	0.50
Choline chloride, 60%	0.25	0.24
Vitamin-mineral premix^2^	1.00	1.00
Calculated Provisions		
AME, kcal/kg	2,846	3,097
Crude protein, %	-	20.0
Crude fat, %	2.00	12.0
Ca, %	0.87	0.87
Non-phytate P, %	0.43	0.43
Total P, %	0.49	0.66
Potassium, %	1.00	1.04
Sodium, %	0.16	0.16
Chloride, %	0.38	0.38

1 Extruded or roasted full fat soybean.

2 Vitamin mineral premix provided per kilogram of diet: vitamin A, 8,800.0 IU; vitamin D3, 3,300.0 IU; vitamin E, 40.0 IU; vitamin B12, 12.0 mg; vitamin K3, 3.3 mg; niacin, 50.0 mg; choline, 1,200.0 mg; folic acid, 1.0 mg; biotin, 0.22 mg; pyridoxine, 3.3 mg; thiamine, 4.0 mg; calcium pantothenic acid, 15.0 mg; riboflavin, 8.0 mg; manganese, 70.0 mg; zinc, 70.0 mg; iron, 60.0 mg; iodine, 1.0 mg; copper, 10 mg; and selenium, 0.3 mg (DSM Nutritional Products Canada Inc., Ayr, ON, Canada).

### Experimental Procedures

A total of 400-day-old male broiler chicks (Ross 708) were procured from a commercial hatchery (Maple Leaf Foods, New Hamburg, ON, Canada) and placed in cages (10 chicks per cage). Each cage (76 cm width, 51 cm depth, and 56 cm height) was equipped with 2 nipple drinkers connected to a common water line supplying the whole room and an independent trough feeder (70 cm length, 8.5 cm width, and 9 cm depth). There were 2 tiers of cages, each with independent lighting. Lighting schedule was 23D:1D at 100 lux on d 1, 12L:12D at 30 lux on d 4 until wk 2, and 8L:16D at 30 lux thereafter. The room temperature was set at 32°C and gradually brought down to 29°C by d 13. The chickens were fed a commercial starter diet (Floradale Feed Mill Limited, Floradale, ON, Canada) for the first 13 d of life. The corn and soybean meal based commercial starter had 22.8, 4.7, 2.8, 0.97, and 0.62% crude protein, crude fat, crude fiber, Ca, and P, respectively.

On d 14, feed was removed for 3 h, birds weighed on a pen basis, and 5 diets (NFD and 4 test diets EFFSB or RFFSB without or with MES) were assigned based on pen weight to give 8 replicates cages per diet. The birds had free access to the diets and water throughout the experimental period. Between d 17 and 20 of age, excreta samples were collected via collection trays installed underneath the cages to determine AMEn. On d 21, all birds were euthanized by cervical dislocation. The contents of the lower half (2–3 cm from cecal junction) to Meckel's diverticulum, the ileum content was expressed by gentle flushing with distilled water. Digesta from birds within a cage were pooled, resulting in 8 samples per dietary treatment, and frozen (−20°C) immediately after collection until required for analyses.

### Sample Processing and Laboratory Analyses

The ileal digesta and excreta samples were freeze-dried and along with FFSB and diet samples, finely ground with a coffee grinder (CBG5 Smart Grind, Applica Consumer Products Inc., Shelton, CT) and thoroughly mixed for chemical analyses. Samples of FFSB, diets, and excreta were analyzed for DM, gross energy, CP, crude fat, Ca, and P. Dry matter determination was carried out according to standard procedures method 930.15 (AOAC, 2005). Gross energy was determined using a bomb calorimeter (IKA Calorimeter System C 6000; IKA Works, Wilmington, NC). All samples were analyzed for N by the combustion method 968.06 (AOAC, 2005) using a CNS-2000 carbon, N, and sulphur analyzer (LECO Corporation, St. Joseph, MI). The CP values were derived by multiplying the assayed N values by a factor of 6.25. Crude fat content was determined using ANKOM XT 20 Extractor (Ankom Technology, Fairport, NY). The samples were wet acid digested with nitric and perchloric acid mixture (AOAC, 2005; method 968.08) and concentrations of Ca and P read on an inductively coupled plasma mass spectrometer (Varian Inc, Palo Alto, CA). The NDF content in FFSB, diets and excreta samples was determined according to (Van Soest et al., 1991) using α-amylase (Sigma No. A3306, Sigma Chemical Co., St. Louis, MO) and sodium sulfite, and corrected for ash concentration adapted for Ankom 200 Fiber Analyzer (Ankom Technology). Titanium content in diets, ileal digesta, and excreta was measured on a UV spectrophotometer following the method of (Myers et al., 2004). For AA analyses, FFSB, diets, and ileal digesta samples were prepared by acid hydrolysis according to AOAC (2005, method 982.30). Briefly, about 100 mg of each sample was digested in 4 mL of 6 N HCl for 24 h at 110°C, followed by neutralization with 4 mL of 25% (wt/vol) NaOH and cooled to room temperature. The mixture was then equalized to 50 mL volume with sodium citrate buffer (pH 2.2) and analyzed using Ultra performance liquid chromatography (**UPLC**, Waters corporation, Millford, CA). Samples for analysis of sulfur containing AA (Methionine and Cysteine) were subjected to performic acid oxidation prior to acid hydrolysis. Tryptophan was not determined. The FFSB samples were further tested for TI, urease activity and KOH protein solubility. TI activity, defined as the number of TI units per milligram of the sample, was determined according to (2017). Urease activity and KOH protein solubility in a commercial laboratory (Midwest Laboratories Inc., Omaha, NE). The enzyme recovery (phytase, protease, xylanase, and β-glucanase) in the diets was analyzed at DSM Nutritional Products laboratories (Belvidere, NJ).

### Calculations and Statistical Analysis

The SID of AA and AR of components were calculated according to Adeola et al. (2016). The AR of GE in FFSB samples was calculated using substitution method (Woyengo et al., 2010; Mwaniki and Kiarie, 2018) with NFD as the basal diet using the following equation:

(1) $$D_{A}=\;D_{B}+\;\frac{\left(D_{D}-D_{B}\right)}{P_{A}}$$

where D_A_ = AR of GE (%) in test sample; D_B_ = retention of AR in NFD; D_D_ = AR of component in test sample containing diet; and P_A_ = proportion (decimal percentage) of test sample in test sample containing diet. Where test sample can be EFFSB or RFFSB without or with MES. The AME contents for EFFSB or RFFSB without or with MES were calculated using measured AME value of the test diets (**AMEtd**) and proportions of other (corn starch, sucrose, and soy oil) energy yielding ingredients (poeyi) and test ingredients (poti) in the diet. Thus,

AMEti=[AMEtd−(AMEnfdxpoeyi)]/poti

where, AMEti is AME of test ingredient (EFFSB or RFFSB without or with MES) and AMEnfd is AME of NFD diet. The AMEtd and AMEnfd were determined according to Woyengo et al. (2010). The AMEn values for the test ingredients were calculated by correcting AMEti for retained nitrogen.

Data were analyzed using PROC MIXED procedure of SAS (SAS Inst. Inc., Cary, NC). The model contained the fixed effects of FFSB processing, MES and associated interactions and the random effect of cage. An alpha level of 0.05 was used to determine statistical significance, and treatments were compared using Tukey's test.

## RESULTS AND DISCUSSION

The chemical analyses of the FFSB samples and experimental diets are shown in Table 2. The concentration of CP, crude fat, GE, AA in FFSB samples was comparable to values reported for full fat soybean (NRC, 2012; Ravindran et al., 2014a; Woyengo et al., 2014; Park et al., 2017; Kiarie et al., 2020). TIs are the most important antinutritional factors present in raw soybeans, and thermal processing methods are optimized for its destruction (NRC, 2012). However, under or excessive heating may negatively impact the meal quality and nutritive value (Araba and Dale, 1990). Urease index (an indicator of underheating) and protein solubility (indicator for overheating) are typical tests run to evaluate the extent of thermal processing (Ravindran et al., 2014b; García-Rebollar et al., 2016). The general recommendations are that adequately heat-processed soy products for animal feeds should have TI of 1.75 to 2.50 TIU/mg, urease index of 0.10 pH unit change or below and KOH-protein solubility of between 70 and 85% (Araba and Dale, 1990; Van Eys, 2012). The TI was 2.0 and 3.0 TIU/mg, KOH protein solubility was 83.3 and 79.3%, urease index was 0.02, and 0.02 pH unit rise for EFFSB and RFFSB, respectively (Table 2). The TI values were within the range of 2.74 to 3.28 TIU/mg observed in four samples of heat processed FFSB (unknown processing method) samples sourced from commercial feed mills in South East Asia (Ravindran et al., 2014a). The four samples exhibited urease index of 0.02 to 0.30 pH rise and KOH-protein solubility of 63.1 to 81.1% (Ravindran et al., 2014a). The assayed phytase, protease, xylanase, and β-glucanase in EFFSB were 1,955 FYT/kg, 8,324 PROT/kg, 568 U/kg, and 195 U/kg, respectively. Corresponding values for RFFSB were 2,190 FYT/kg, 8,925 PROT/kg, 391 U/kg, and 190 U/kg, respectively. The enzymes recovery levels were within the acceptable industry standards (Bedford, 2018)

**Table 2:** Analyzed chemical composition of the ingredients and experimental diets.

	Ingredients		Diets^1^		
	Extruded	Roasted	N-free	EFFSB	RFFSB
Dry matter, %	91.7	92.7	92.6	91.6	93.3
Gross energy, kcal/kg	5,173	5,214	3,471	4,298	4,347
Crude fat, %	19.2	19.6	1.84	11.3	9.57
Neutral detergent fiber, %	12.7	12.0	1.65	12.3	13.1
Crude protein, %	37.3	35.1	0.75	19.2	21.0
Calcium, %	0.17	0.24	0.85	0.74	0.66
Phosphorous, %	0.62	0.60	0.33	0.59	0.60
Indispensable AA, %					
Arg	2.63	2.58	0.02	1.39	1.46
His	0.98	0.94	0.01	0.59	0.69
Ile	1.68	1.64	0.02	0.91	1.11
Leu	2.72	2.70	0.05	1.59	1.93
Lys	2.24	2.22	0.02	1.04	1.24
Met	0.52	0.52	0.01	0.19	0.23
Phe	1.82	1.79	0.03	1.28	1.55
Thr	1.46	1.43	0.02	0.71	0.83
Val	1.73	1.72	0.03	0.99	1.13
Dispensable AA, %					
Ala	1.53	1.54	0.03	0.81	0.96
Asp	4.01	3.97	0.04	2.10	2.23
Cys	0.53	0.55	0.00	0.13	0.16
Glu	6.34	4.84	0.64	2.66	2.63
Gly	1.55	1.53	0.02	1.01	1.08
Pro	1.81	1.76	0.03	1.24	1.41
Ser	1.89	1.81	0.02	0.96	1.16
Tyr	1.28	1.28	0.02	0.95	1.10
Soy quality check					
Trypsin inhibitors, mg/g	2.00	3.00	-	-	-
KOH, %	83.3	79.3	-	-	-
Urease activity, pH rise unit	0.02	0.02	-	-	-
Lys to CP ratio, %	7.06	7.06	-	-	-

1 NFD, nitrogen free, extruded or roasted full fat soybean.

There was no (*P* > 0.05) interaction between processing and MES of SID of AA (Table 3). The processing impact on AA digestibility was such that, among the indispensable AA birds fed EFFSB had higher (*P* ≤ 0.05) SID of Arg (93.2 vs. 87.8%), Ile (87.7 vs. 83.3%), Lys (87.1 vs. 78.1%), and Met (93.5 vs. 90.3%) than the birds fed RFFSB. Birds fed EFFSB also showed higher (*P* ≤ 0.05) SID of Asp, Glu, Gly, and Ser relative to birds fed RFFSB. Among the indispensable AA, Thr (84.5%), and Lys (78.1%) were the least digestible in EFFSB and RFFSB, respectively. However, the observed SID values for the indispensable AA in the present study were within the range of 71.1 % for Thr to 84.8% for Arg reported for FFSB fed to broiler chickens (Park et al., 2017). A range of 74.1% for Thr to 89.8% for Arg was observed for SID of indispensable AA in four heat processed FFSB samples fed to broiler chickens (Ravindran et al., 2014a). A database of SID of indispensable AA in FFSB reported a range of 83% for Thr to 87% for Met and His (Evonik, 2016). Higher values for SID of indispensable AA in heat processed FFSB fed broiler chickens have also been reported. For example, a range of 91.3 for Leu to 95.3% for Phe was reported for FFSB cooked in a hydrothermal reactor followed by expansion (Valencia et al., 2009). The SID of AA of the FFSB samples tested in the present study were within the range of values reported for thermally processed FFSB (Ravindran et al., 2014a; Evonik, 2016; Park et al., 2017). The differences between EFFSB and RFFSB in digestibility of AA might be partly attributed to processing. A proper processing technique ought to reduce TI to acceptable levels with no detrimental impact on the nutritional attributes. The observed TI values in FFSB samples in the present study were well below the tolerance level of 4.1 TIU/mg reported for broilers chickens (Ravindran et al., 2014a). Interestingly, broilers fed FFSB cooked in hydrothermal reactor followed by expansion had much higher SID of Lys (93.3%) than FFSB in the present study yet that sample had higher TI (8.2 TIU/mg) concentration (Valencia et al., 2009). Four samples of heat processed FFSB (TI, range 2.74–3.28 TIU/mg) had SID of Lys values ranging from 79.7 to 85.1% (Ravindran et al., 2014a). A SID of Lys of 78.2% was observed in broiler chickens fed FFSB (heat processing regimen not specified) containing 5.50 TIU/mg (Park et al., 2017).

**Table 3:** Effects of full fat soybeans (FFSB) processing method and multi enzymes supplement on standardized ileal digestibility of amino acids in 21-day-old broiler chickens-FFSBM-corn starch diet^1^.

Processing^2^ MES^3^	EFFSB		RFFSB			Processing		MES		SEM	*P*-values		
	−	+	−	+	SEM	EFFSB	RFFSB	−	+		Processing	MES	Processing × MES
Indispensable													
Arg	92.2	94.1	86.9	88.8	1.35	93.2^a^	87.8^b^	89.5	91.4	0.95	<0.01	0.174	0.970
His	85.5	91.4	85.2	86.5	2.29	88.4	85.8	85.3	88.9	1.62	0.269	0.132	0.333
Ile	86.4	89.0	83.1	83.6	2.07	87.7^a^	83.3^b^	84.8	86.3	1.47	0.048	0.473	0.622
Leu	85.6	89.6	84.2	84.4	2.16	87.6	84.3	84.9	87.0	1.53	0.144	0.343	0.408
Lys	84.4	89.7	77.1	81.1	2.08	87.1^a^	79.2^b^	80.8^b^	85.5^a^	1.47	0.002	0.050	0.804
Met	93.2	93.8	88.9	91.7	1.29	93.5^a^	90.3^b^	91.0	92.8	0.91	0.022	0.194	0.420
Phe	88.1	91.8	87.2	86.9	1.77	90.0	87.0	87.6	89.3	1.26	0.112	0.357	0.279
Thr	83.2	85.9	81.1	82.9	2.52	84.5	82.0	82.1	84.4	1.78	0.325	0.374	0.874
Val	86.0	89.2	84.2	85.0	2.30	87.6	84.6	85.1	87.1	1.63	0.209	0.394	0.606
Dispensable													
Ala	85.7	88.8	83.3	84.1	2.20	87.2	83.7	84.5	86.4	1.56	0.123	0.384	0.623
Asp	87.6	88.8	75.3	78.0	2.19	88.2^a^	76.7^b^	81.4	83.4	1.55	<0.01	0.380	0.723
Cys	79.7	78.2	74.8	82.5	2.63	78.9	78.6	77.2	80.3	1.86	0.919	0.256	0.097
Glu	87.0	88.3	81.3	82.0	2.90	87.7^a^	81.7^b^	84.1	85.2	2.06	0.051	0.718	0.917
Gly	84.8	86.3	80.9	80.5	2.05	85.5^a^	80.7^b^	82.8	83.4	1.45	0.027	0.795	0.638
Pro	85.6	90.9	86.8	83.8	2.39	88.2	85.3	86.2	87.3	1.69	0.234	0.628	0.097
Ser	87.4	87.6	81.6	83.3	2.06	87.5^a^	82.5^b^	84.5	85.5	1.46	0.023	0.622	0.724
Try	84.6	88.7	85.4	84.9	2.00	86.7	85.2	85.0	86.8	1.42	0.461	0.396	0.264

1 Calculated by correcting values for apparent digestibility for basal endogenous losses from birds fed N-free diets: 0.35, 1.38, 0.33, 0.47, 0.31, 0.13, 0.27, 0.67, 0.49, 0.39, 0.64, 0.23, 0.92, 0.42, 0.58, 0.65, and 0.21 g/kg DM intake for Arg, His, Ile, Leu, Lys, Met, Phe, Thr, Val, Ala, Asp, Cys, Glu, Gly, Pro, Ser, and Tyr, respectively.

2 Extruded or roasted full fat soybean.

3 Multienzyme supplement supplied main activities of phytase, protease, xylanase, β-glucanase at 2,200 FYT/kg, 8,300 PRO/kg, 600 U/kg, and 200 U/kg of feed, respectively (Victus, DSM Nutritional Products Inc., Parsippany, NJ).

a-c Means assigned different letters within a factor of analysis (processing, MES, and their interactions) are significantly different, *P* < b0.05

The difference (∼12%) in SID of Lys in FFSB samples suggested other factors other than TI could have been at play. Although RFFSB sample had lower KOH-protein solubility values than EFFSB sample, this value was within the suggested range of >70% (Araba and Dale, 1990; Van Eys, 2012), indicating no evidence of overheating/cooking. It is plausible prolonged heat treatment may have rendered Lys unavailable in RFFSB sample through the formation of Maillard reaction products. However, the concentration of starch and sugars in soybean seeds has been reported to be less than 10% (NRC, 2012; Ravindran et al., 2014a). Moreover, the formation of Maillard reactions in soybean processing has been demonstrated during co-application of higher temperature regimens (>125°C) and steam (Qin et al., 1998; González-Vega et al., 2011). Heating soybean meal to 125°C in dry heating (akin to roaster used for RFFSB) did not result in the formation of Maillard products, and there was no detrimental impact on Lys digestibility in pigs (González-Vega et al., 2011). Research in ruminants has shown that processing protein feedstuffs increase the concentration of neutral and acid detergent insoluble nitrogen (**NDIN** and **ADIN)** with negative consequences in CP utilization (Mustafa et al., 2001; Machacek and Kononoff, 2009). For example, samples of EFFSB were observed to have ADIN of 2.8 g/kg DM and effective degradability (**ED**) of CP of 63.9%, whereas toasted FFSB had ADIN of 5 g/kg DM and ED of 57.3% (González et al., 2002). It can be postulated that some CP/AA in RFFSB may have been indigestible due to being fiber bound; however, this parameter was not measured in this study.

Components such as phytate, indigestible fiber and protein complexes are inherently present in feedstuffs but can also be created or enhanced, for example, NDIN and ADIN by the processing regimens (González et al., 2002; Kiarie et al., 2016b; Kiarie and Mills, 2019; Kiarie, 2020). An evaluation of 55 soybean meal samples (CP 44–48%) from commercial feed mills demonstrated that variation in amino acids and energy digestibility in broiler chickens was not correlated with processing quality checks (TI, urease index, and KOH-protein solubility) or concentration of CP but was highly negatively correlated with the concentration of fiber and ash (Ravindran et al., 2014b). Application of feed enzymes in monogastric feeding programs targets anti-nutritional and indigestible components in feedstuffs (Bedford and Schulze, 1998; Adeola and Cowieson, 2011; Slominski, 2011; Ravindran, 2013; Kiarie et al., 2016b). Therefore, it is relevant that birds fed MES with phytase, protease and fiber degrading enzymes had higher (85.5 vs. 80.8%; *P* = 0.050) SID of Lys than birds fed non-MES diet (Table 3). Similarly, supplemental protease improved the SID of CP and Lys in broiler chickens fed raw FFSB (Erdaw et al., 2017a). An enzyme supplement containing pectinase, cellulase, mannanase, xylanase, β-glucanase, and galactanase improved SID values of CP, Leu, Lys, Met + Cys and Thr in extruded FFSB fed to finishing pigs (Ayoade et al., 2012). Although numerically, the SID of Arg, His, Ile, Leu, Met, Phe, Thr, and Val for birds fed MES were 2.0, 4.2, 1.8, 2.5, 2.0, 1.9, 2.8, and 2.4%, respectively higher than for birds not fed MES. The corresponding differences for Ala, Asp, Cys, Glu, Gly, Pro, Ser and Tyr were 2.2, 2.5, 4.0, 1.3, 0.7, 1.3, 1.2, and 2.1%, respectively (Table 3). The same supplemental MES did not affect the SID of Lys in pigs fed RFFSB (Kiarie et al., 2020). This may be linked to differences between digestive capacities in pigs and poultry. For example, a comparative study indicated that pigs digested more amino acids in FFSB than broiler chickens (Park et al., 2017). Indeed, in our previous pig study, the observed SID of Lys of RFFSB was 82.6% (Kiarie et al., 2020). As such benefits of supplemental feed enzymes are more pronounced in poultry (Bedford and Schulze, 1998).

An interaction (*P* = 0.04) was observed between processing and MES on AR of DM such that MES improved AR of DM in birds fed RFFSB only (Table 4). There was no (*P* > 0.05) interaction between processing and MES on AR of crude fat, CP, and NDF (Table 4). The main effects were such that birds fed EFFSB had higher (*P* ≤ 0.01) AR of crude fat (90.5 vs. 63.3%), NDF (26.1 vs. 19.1%) but lower (*P* < 0.01) AR of CP (59.5 vs. 53.2%) than birds fed RFFSB. Birds fed MES showed higher (*P* = 0.028) AR of CP (57.5 vs. 55.2%) than non-MES birds. There was an interaction (*P* ≤ 0.030) between processing and MES on AR of GE, Ca, and P (Table 4). In this context, MES improved the AR of GE in RFFSB and not in EFFSB. Birds fed EFFSB with MES showed higher AR of Ca and P than birds fed all the other diets. Within birds fed RFFSB, birds fed MES had higher AR of Ca and P than non-MES birds. In essence, MES improved Ca and P digestibility by 15.3 and 32.5% in EFFSB and 23.5 and 31.8% in RFFSB, respectively extending well established concept that phytase increased phytate degradation in oilseeds (Kiarie et al., 2016a). Pigs fed RFFSB supplemented with the same MES used in the present study showed ∼5% higher Ca and P digestibility than pigs fed RFFSB without MES (Kiarie et al., 2020).

**Table 4:** Effects of full fat soybean (FFSB) processing method and multienzymes supplement (MES) on apparent retention of components in 21-day-old broiler chickens fed corn starch diet.

Processing	MES^1^	Dry matter	Crude fat	Crude protein	NDF	Gross energy	Ca	P
Extruded	−	71.7^a^	90.9	52.4	26.9	78.9^a^	45.9^b^	32.0^bc^
Extruded	+	71.7^a^	90.2	54.0	25.3	78.6^a^	52.9^a^	42.4^a^
Roasted	−	65.8^c^	61.1	58.0	17.7	69.5^c^	37.0^c^	26.7^c^
Roasted	+	68.7^b^	65.5	61.0	20.5	72.2^b^	45.7^b^	35.2^b^
SEM		0.626	1.98	0.99	2.38	0.64	1.40	1.58
Processing								
Extruded		71.7	90.5^a^	53.2^b^	26.1^b^	78.8	49.4	37.2
Roasted		67.5	63.3^b^	59.5^a^	19.1^a^	70.9	41.4	31.0
MES								
-		68.8	76.0	55.2^b^	22.3	74.2	49.3	29.1
+		70.2	77.8	57.5^a^	22.9	75.4	41.3	39.1
SEM		0.471	1.40	0.70	1.69	0.453	0.990	1.11
Processing		<0.01	<0.01	<0.01	0.007	<0.01	<0.01	<0.01
MES		0.043	0.363	0.028	0.801	0.061	<0.01	<0.01
Processing × MES		0.042	0.215	0.510	0.373	0.030	0.006	0.036

1 Multienzyme supplement supplied main activities of phytase, protease, xylanase, β-glucanase at 2,200 FYT/kg, 8,300 PRO/kg, 600 U/kg, and 200 U/kg of feed, respectively (Victus, DSM Nutritional Products Inc., Parsippany, NJ).

a-c Means assigned different letters within a factor of analysis (processing, MES, and their interactions) are significantly different, *P* < 0.05.

Energy utilization in feedstuffs is critical in practical poultry nutrition. The merits and demerits of numerous protocols for determining metabolizable energy content in poultry feedstuffs were recently reviewed (Wu et al., 2020). The current study investigated energy utilization in EFFSB and RFFSB using semi-purified diets. This protocol may have limitations related to the assumption of additivity of energy of individual ingredients (Wu et al., 2020). The AR of GE, AME, and AMEn for FFSB are shown in Table 5. There was an interaction (*P* ≤ 0.030) between processing and MES on AR of GE, AME, and AMEn such that MES improved utilization of energy in RFFSB and not in EFFSB. Previous research indicated that reduced energy use in full-fat oil seeds is due to oil encapsulation by fibrous the cell wall polysaccharides (Meng et al., 2006; Slominski et al., 2006). Although we did not observe interaction (*P* > 0.05) between processing and MES on AR of crude fat, it was noteworthy that birds fed RFFSB with MES retained ∼7% more crude fat than birds fed RFFSB without MES. Moreover, the supplemental MES used in the present study resulted in a 13% improvement in apparent tract digestibility of GE in RFFSB and soybean meal fed to pigs linked to a 28.6% improvement in crude fat digestibility (Kiarie et al., 2020). While FFSB samples had comparable NDF, it is plausible that processing of RFFSB may have resulted in indigestible fiber-protein complexes such as NDIN and ADIN that are amenable to supplemental MES, leading to increased release of energy yielding substrates. Moreover, the enzyme composite tested in the present study allowed for the sustained growth performance in nursery pigs fed diets with various nutrient and ingredient reductions (Tsai et al., 2017). The MES also increased ileal digestible energy and overcame reductions in energy, P and amino acid contents in corn and SBM diet (Jasek et al., 2015) and significantly improved broiler growth performance (Ward et al., 2014; Jasek et al., 2015; Ward et al., 2015, 2020).

**Table 5:** Effects of full fat soybeans (FFSB) processing method and multi enzymes supplement (MES) on apparent gross energy retention and metabolizable energy in FFSB samples fed to 21-day-old broiler chickens^1^.

Processing	MES^2^	Gross energy, %	AME, kcal/kg DM	AMEn, kcal/kg DM
Extruded	No	69.9^a^	4,048^a^	3,904^a^
Extruded	Yes	70.2^a^	4,055^a^	3,906^a^
Roasted	No	58.3^b^	3,463^b^	3,337^b^
Roasted	Yes	63.6^c^	3,701^c^	3,561^c^
SEM		1.04	47.9	48.8
Processing				
Extruded		70.0	4,052	3,905
Roasted		60.9	3,582	3,449
MES				
No		64.1	3,755	3,620
Yes		66.9	3,878	3,733
SEM		0.71	32.8	31.6
*P*-value				
Processing		<0.01	<0.01	<0.01
MES		0.061	0.011	0.015
Processing × MES		0.030	0.016	0.016

1 Calculated by difference method considering energy yielding components supplied by FFSBM, corn starch, sucrose, and soy oil (Mwaniki and Kiarie, 2018).

2 Multienzyme supplement supplied main activities of phytase, protease, xylanase, β-glucanase at 2,200 FYT/kg, 8,300 PRO/kg, 600 U/kg, and 200 U/kg of feed, respectively (Victus, DSM Nutritional Products Inc., Parsippany, NJ).

a-c Means assigned different letters within a factor of analysis (processing, MES, and their interactions) are significantly different, *P* < 0.05.

An optimally processed FFSB is a valuable feed ingredient for poultry because of high-quality protein and energy in the form of oil. Generally, extrusion is the dominant thermal treatment accepted for producing high quality FFSB with high available energy content due to complete rupture of oil cells compared to other methods (Waldroup, 1982; Ravindran et al., 2014a). Moreover, processing parameters such as temperature, time, pressure, moisture content, and the degree of physical pressure can all be manipulated to optimize meal quality (Kim et al., 2006; Singh et al., 2007). However, variation in processing approaches exists. For example, dry roasting is seen as inexpensive on-farm processing that can be employed in small-to-medium operations that cannot afford an extruder. However, regardless of thermal treatment, the resulting FFSB product must have safe levels of heat labile antinutritional factors without penalty on the nutritive value. We demonstrated that EFFSB had better nutritive value in terms of digestibility of AA and energy than RFFSB in broiler chickens, partly reflecting the impact of processing and compositional differences on nutrient utilization. Utility of feed enzyme supplement containing phytase, protease, and fiber degrading enzymes improved lysine and minerals utilization in both products. However, the enzyme improved energy utilization in roasted FFSB, suggesting value in enhancing utilization of nutrients in feedstuffs subject to varied processing.
